# Supplementary material for: Anthrax toxin translocation complex reveals insight into the lethal factor unfolding and refolding mechanism
Source: Sci Rep. 2021 Jun 22;11:13038. doi: 10.1038/s41598-021-91596-3 (PMC8219829; doi:10.1038/s41598-021-91596-3)
Supplement: Supplementary file 1 — Supplementary Information. [file 41598_2021_91596_MOESM1_ESM.pdf]

SUPPLEMENTARY INFORMATION

Anthrax Toxin Translocation Complex Reveals insight into the Lethal Factor Unfolding and Refolding Mechanism

Alexandra J Machen, Mark T Fisher, Bret D Freudenthal

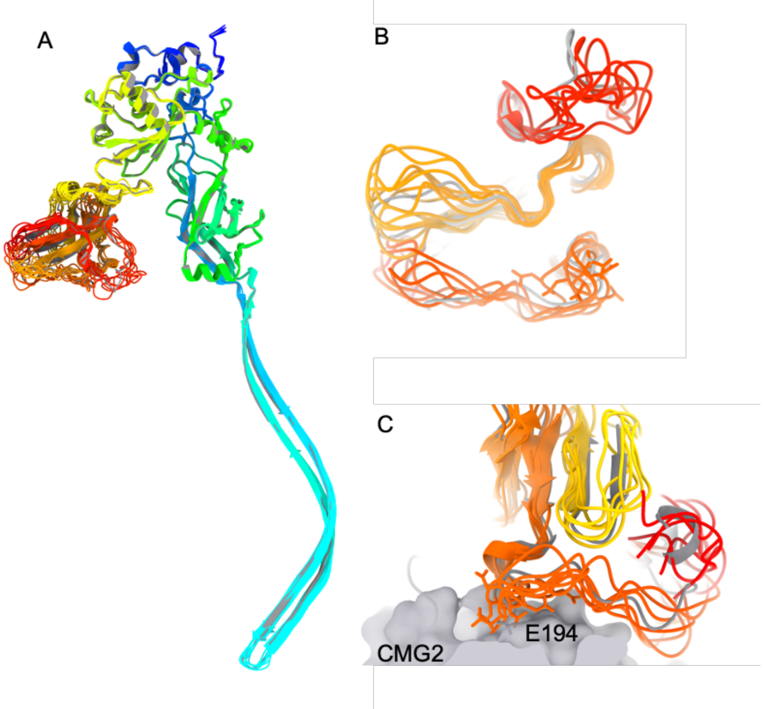

Figure S1. Comparison of PA_{pore} protomers. (A) PA_{pore} protomer chains are shown in rainbow N to C termini for chains A-G. The receptor binding domain is shown in yellow, red, and orange. (B) Bottom up view of receptor binding domain with $PA_{prepore}$ receptor binding domain shown in grey (PDB 1T6B). (C) Comparison of PA_{pore} protomers to $PA_{prepore}$ bound to receptor CMG2 (grey).

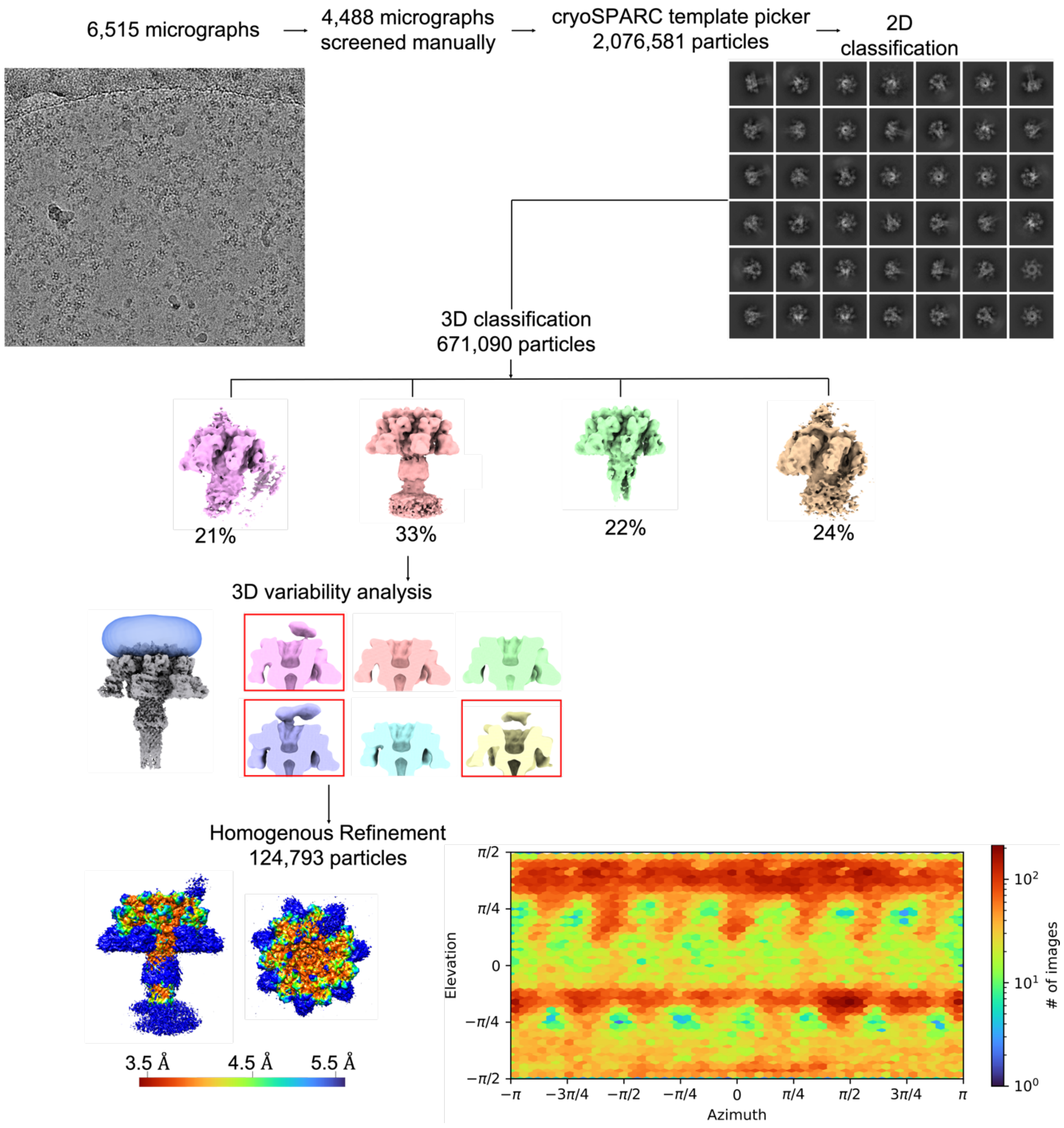

Figure S2. Single particle analysis of anthrax toxin translocating complexes. After micrograph curation and 2D classification, 'good' particles were subjected to 3D classification (four classes) followed by 3D variability analysis on particles that contained intact β barrel. 3D variability analysis focused on potential LF_N binding sites (blue mask) above PA_{pore} (grey). Results of 3D variability analysis are shown in pastels filtered to 20 Å (pink, orange, green, purple, blue, yellow) with potential LF_N density containing maps boxed in red. 3.3 Å refinement of PA_{pore} with intact β barrel and potential LF_N density is shown in grey (FSC cutoff 0.143). Euler angle distribution of particles shows diverse particle orientations.

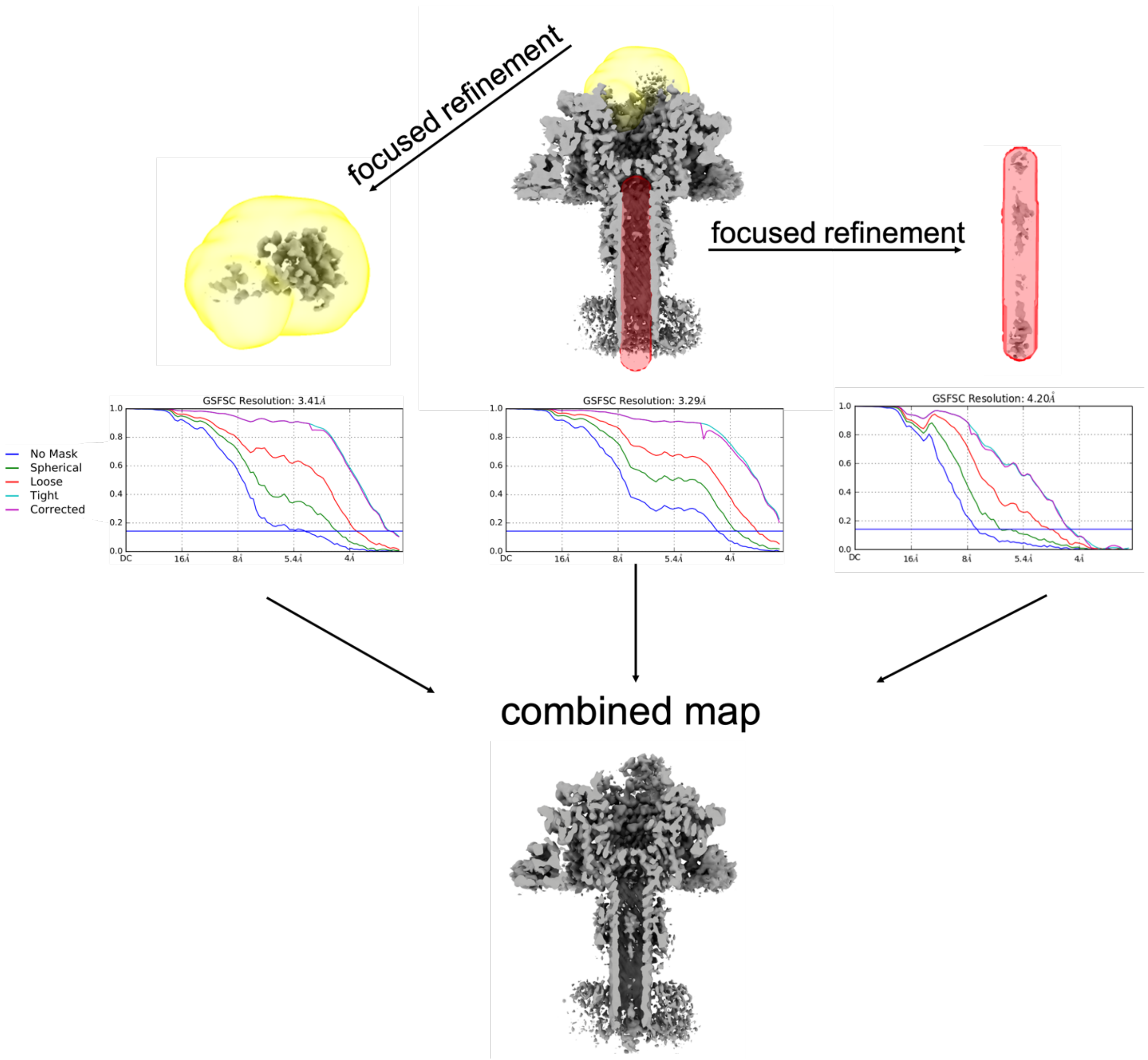

Figure S3. Density modification of cryoEM map. Focused refinement and density modification of non-uniform refined cryoEM map were perfomed in cryoSPARC and Phenix, respectively. Density for LF_N above PA_{pore}, PA_{pore}, and β barrel interior were combined using Phenix combine focus maps. Resolution was determined for each density prior to combining using FSC cutoff of 0.143.

Supplementary Table 1. cryoEM Data Collection and Processing Statistics

**Data collection and processing**	
Magnification	130,000X
Voltage (kV)	300
Electron exposure ($e^-/Å^2$)	1.79 per frame
Defocus range (μm)	1-3um
Pixel size (Å)	0.535
Symmetry imposed	C1
Initial particle images (no.)	2076581
Final particle images (no.)	122651
Map resolution (Å)	3.3
FSC threshold	0.143
**Refinement**	
Initial model used (PDB ID)	6PSN
Model resolution (Å)	3.3
FSC threshold	0.143
**Model composition**	
Nonhydrogen atoms	32826
Protein residues	4154
Ligands	14
B factors ($Å^2$)	
Protein	396
Ligand	245
**r.m.s. deviations**	
Bond Length (Å) (# > 4σ)	0.009 (2)
Bond Angles (°) (# > 4σ)	1.317 (40)
**Validation**	
MolProbity score	2.76
Clashscore	26
Poor rotamers (%)	4.0
**Ramachandran plot**	
Favored (%)	94.18
Allowed (%)	5.25
Disallowed (%)	0.58
